# Comparative Outcomes of Empagliflozin to Dapagliflozin in Patients With Heart Failure

**DOI:** 10.1001/jamanetworkopen.2024.9305

**Published:** 2024-05-02

**Authors:** Katherine L. Modzelewski, Alexandra Pipilas, Nicholas A. Bosch

**Affiliations:** 1 Section of Endocrinology, Diabetes, Nutrition and Weight Management, Department of Medicine, Boston University Chobanian and Avedisian School of Medicine, Boston, Massachusetts; 2 Cardiovascular Medicine, Department of Medicine, Boston University Chobanian and Avedisian School of Medicine, Boston, Massachusetts; 3 The Pulmonary Center, Boston University Chobanian and Avedisian School of Medicine, Boston, Massachusetts

## Abstract

**Importance:**

Sodium-glucose cotransporter-2 (SGLT2) inhibitors have been shown to have benefits when used in patients with heart failure. The comparative outcomes of SGLT2 inhibitors relative to each other has not been well defined and may impact medication selection.

**Objective:**

To determine the comparative outcomes of empagliflozin and dapagliflozin on reducing the composite of all-cause mortality and hospitalizations in patients with heart failure.

**Design, Setting, and Participants:**

This multicenter retrospective cohort study included patients with heart failure from August 18, 2021, and December 6, 2022, in the TriNetX Research Collaborative, a centralized database of deidentified electronic medical record data from a network of 81 health care organizations. Eligible patients had a diagnosis of heart failure, had never received an SGLT2 inhibitor previously, and were newly started on empagliflozin or dapagliflozin. Patients were followed up for 1 year.

**Exposure:**

Initiation of dapagliflozin or empagliflozin.

**Main Outcomes and Measures:**

The primary outcome was the time to the composite of all-cause mortality or hospitalization between study days 1 to 365. Kaplan-Meier analyses, hazard ratios (HRs), and 95% CIs were used to assess the primary outcome.

**Results:**

Among 744 914 eligible patients, 28 075 began empagliflozin (15 976 [56.9%]) or dapagliflozin (12 099 [43.1%]). After nearest-neighbor matching for demographics, diagnoses, and medication use, there were 11 077 patients in each group. Of patients who received empagliflozin, 9247 (57.9%) were male, 3130 (19.6%) were Black individuals, and 9576 (59.9%) were White individuals. Similarly, of those who received dapagliflozin, 7439 (61.5%) were male, 2445 (20.2%) were Black individuals, and 7131 (58.9%) were White individuals. Patients receiving empagliflozin were less likely to experience the composite of all-cause mortality or hospitalization compared with those initiated on dapagliflozin (3545 [32.2%] vs 3828 [34.8%] events; HR, 0.90 [95% CI, 0.86-0.94]) in the year following SGLT2 inhibitor initiation and less likely to be hospitalized (HR, 0.90 [95% CI, 0.86-0.94]). All-cause mortality did not differ between exposure groups (HR, 0.91 [95% CI, 0.82-1.00]). There was no difference in mean hemoglobin A_1c_ or adverse events between groups.

**Conclusions and Relevance:**

In this cohort study, patients who initiated empagliflozin were less likely to experience the composite of all-cause mortality or hospitalization compared with patients who started dapagliflozin. Additional studies are needed to confirm these finding.

## Key Points

**Question:**

What are the comparative outcomes of empagliflozin and dapagliflozin in reducing all-cause mortality and hospitalizations in patients with heart failure?

**Findings:**

In this cohort study of 28 075 patients with heart failure naive to sodium-glucose cotransporter-2 inhibitor therapy, patients who started using empagliflozin were less likely to experience the primary outcome of composite of all-cause mortality or hospitalization compared with those who started using dapagliflozin.

**Meaning:**

These findings suggest that further studies are needed to clarify mechanisms that could explain outcomes of empagliflozin and dapagliflozin in patients with heart failure.

## Teaser

This cohort study compares all-cause mortality and hospitalizations in patients with heart failure receiving empagliflozin vs dapagliflozin.

## Introduction

The sodium-glucose cotransporter-2 (SGLT2) inhibitors empagliflozin and dapagliflozin reduce cardiovascular death and heart failure hospitalizations in patients with heart failure.^1^ However, cardiac medications within the same class may not all have the same benefit. For example, carvedilol reduces mortality by 16% relative to metoprolol in patients with heart failure,^2^ and chlorthalidone is more potent than hydrochlorothiazide in the treatment of essential hypertension.^3^ In patients with diabetes, empagliflozin may be associated with greater weight loss, reduction of blood pressure, and reduction of cholesterol compared with dapagliflozin.^4^ In patients with heart failure, a single center retrospective study suggested that empagliflozin may be associated with improvements in left ventricular ejection fraction and functional status compared with dapagliflozin.^5^ However, the outcomes of empagliflozin vs dapagliflozin on clinically important patient-centered outcomes for patients with heart failure is unclear. In this multicenter retrospective cohort study, we sought to compare the composite outcome of all-cause mortality and hospitalization between those initiated on empagliflozin vs dapagliflozin in patients with heart failure.

## Methods

This study follows the Strengthening the Reporting of Observational Studies in Epidemiology (STROBE) reporting guideline. Informed consent and review were not required because this study was designated to not be human participant research by the institutional review board of Boston University.

### Data Source and Cohort

We used the TriNetX Research Collaborative Network,^6,7^ a network of 81 health care organizations primarily in North America that contribute deidentified electronic medical record data to a central database. We included patients with heart failure (*International Statistical Classification of Diseases and Related Health Problems, Tenth Revision* [*ICD-10*] code: I50x), who had never received SGLT2 inhibitors previously, and were newly started on empagliflozin or dapagliflozin. Patients were included if they met criteria between August 18, 2021 (after publication of the Dapagliflozin Evaluation to Improve the Lives of Patients with Preserved Ejection Fraction Heart Failure, or DELIVER, Trial^8^ and after both SGLT2 inhibitors were approved for heart failure by the US Food and Drug Administration),^9,10^ and December 6, 2022 (to allow all patients to have 1-year of follow-up time). Study day 0 was defined as the day of SGLT2 inhibitor initiation.

### Exposure

The intervention group was defined as initiation of empagliflozin, and the comparator group was defined as initiation of dapagliflozin. Consistent with an intention-to-treat clinical trial, all patients were analyzed according to the SGLT2 inhibitor initially received, regardless of changes in medication use after initiation.

### Outcomes

The primary outcome was the time to the composite of all-cause mortality or hospitalization between study days 1 to 365. Due to limitations in the online TriNetX Query Builder and Analytics platforms, we were unable to determine cause-specific mortality or hospitalizations. Secondary outcomes were all-cause mortality, hospitalization, and last measured hemoglobin A_1c_. Adverse effects were defined as occurrence of urinary tract infection (*ICD-10* code: N39.0) or diabetic ketoacidosis (*ICD-10* codes: E10.1x or E11.1x).

### Covariables

From each patient, we extracted covariables from study days −365 to 0 for inclusion in propensity score models that we thought were likely to confound the association between SGLT2 inhibitor selection and the composite outcome. Covariables included demographics, cardiac and diabetes-related diagnoses and medication use, glomerular filtration rate, hemoglobin A_1c_, natriuretic peptides, left ventricular ejection fraction, and hospitalizations. A complete list of covariables and covariable definitions are included in eTable 1 in Supplement 1.

### Statistical Analysis

Prior to matching, covariables were summarized using mean (SD) and No. (%) as appropriate. We generated propensity scores for empagliflozin vs dapagliflozin initiation using logistic regression and included all covariables in the model. Covariables, except for age, were included in the model as categorical variables. For other variables that were originally continuous (glomerular filtration rate, hemoglobin A_1c_, natriuretic peptides, and left ventricular ejection fraction), values were assigned into clinically relevant categories. This categorization allows for the inclusion of all covariables and patients in the models, even in the setting of multiple measurements or missing data. For example, a patient with no hemoglobin A_1c_ measured in the year prior to study day 0 would be assigned a 0 for all hemoglobin A_1c_ categories, and a patient with multiple hemoglobin A_1c_ measurements in the year prior to study day 0 could potentially be assigned a 1 for multiple hemoglobin A_1c_ categories. After propensity score generation, we used 1:1 greedy nearest neighbor propensity score matching without replacement to create balanced cohorts. Balance was visualized using density curves of the propensity score and formally assessed using absolute standardized mean differences (SMDs) with a SMD of less than 0.1 defining similarity. We then used the Kaplan-Meier method^11^ and log-rank test to compare outcomes between matched groups; hazard ratios (HRs), 95% CIs, and absolute risk differences (95% CI) were also reported. Patients were censored from the Kaplan-Meier analysis on the day after the last fact in their TriNetX record. E-values^12^ were calculated using the observed HRs and a freely available online calculator.^13,14,15^ We repeated analyses in patients with heart failure with reduced ejection fraction (HFrEF) (*ICD-10* codes: I50.2x or I50.4x) and heart failure with preserved ejection fraction (HFpEF) (*ICD-10* codes: I50.3x or I50.4x). For the secondary outcome of last measured hemoglobin A_1c_, patients with no values were excluded from the analysis and the *t* statistic was used to compare mean values between patients who received empagliflozin and dapagliflozin.

Cohort identification and statistical analyses were conducted using the TriNetX Platform^16^ Query Builder and Analytics Functions, respectively, on December 6, 2023. This point-and-click platform allows users to conduct observational studies using curated variable definitions and a limited set of analytic approaches. Kaplan-Meier curves were recreated using R version 4.2.1 (R Project for Statistical Computing). α was set at .05, and all hypothesis tests were 2-sided. We did not account for multiple testing. Thus, secondary outcomes and analyses should be considered hypothesis generating only.

## Results

**Figure 1.**
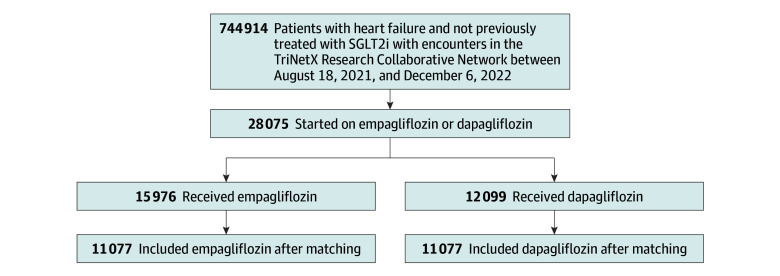
Study Flow Diagram SGLT2 indicates SGLT2i indicates sodium-glucose cotransporter-2 inhibitors.

Among 744 914 patients with heart failure and naive to SGLT2 inhibitor therapy, 28 075 began empagliflozin (15 976 [56.9%]) or dapagliflozin (12 099 [43.1%]) (Figure 1). Prior to matching, the mean (SD) age was 66.4 (13.4) years for participants who received empagliflozin and 63.8 (14.2) years for participants who received dapagliflozin. Of those who received empagliflozin, 9247 (57.9%) were male, 3130 (19.6%) were Black patients, and 9576 (59.9%) were White patients. Similarly, of those who received dapagliflozin, 7439 (61.5%) were male, 2445 (20.2%) were Black patients, and 7131 (58.9%) were White patients (Table). The use of β blockers (SMD, 0.043) and angiotensin converting enzyme inhibitors (SMD, 0.065) inhibitors was similar between prematched groups. The use of angiotensin II inhibitors (7731 [63.9%] vs 8852 [55.4%]; SMD, 0.174) and sacubitril (5471 [45.2%] vs 5258 [32.9%]; SMD, 0.254) was higher in those who received dapagliflozin compared with those who received empagliflozin (Table). Postmatching characteristics (11 007 patients per group) were all similar between groups (Table). The largest difference in covariables after matching was for the use of angiotensin converting enzyme inhibitors (empagliflozin: 2917 [26.5%] vs dapagliflozin: 2982 [27.1%]; SMD, 0.013). Propensity score density curves before and after matching are shown in eFigure 1 in Supplement 1.

**Table.** Characteristics of Patients With Heart Failure in the 12 Months Prior to Empagliflozin or Dapagliflozin Initiation^a^

Characteristic	Patients, No. (%)					
	Prematching			Postmatching		
	Empagliflozin (n = 15 976)	Dapagliflozin (n = 12 099)	SMD	Empagliflozin (n = 11 007)	Dapagliflozin (n = 11 007)	SMD
Age, mean (SD), y	66.4 (13.4)	63.8 (14.2)	0.190	64.8 (13.7)	64.8 (13.8)	0.003
Sex						
Male	9247 (57.9)	7439 (61.5)	0.074	6712 (61.0)	6679 (60.7)	0.006
Female	5930 (37.1)	4319 (35.7)	0.030	3960 (36.0)	3987 (36.2)	0.005
Race						
Asian	520 (3.3)	253 (2.1)	0.072	250 (2.3)	250 (2.3)	0
American Indian or Alaska Native	49 (0.3)	36 (0.3)	0.002	33 (0.3)	33 (0.3)	0
Black or African American	3130 (19.6)	2445 (20.2)	0.015	2194 (19.9)	2201 (20.0)	0.002
Native Hawaiian or other Pacific Islander	177 (1.1)	44 (0.4)	0.087	50 (0.5)	44 (0.4)	0.008
White	9576 (59.9)	7131 (58.9)	0.020	6704 (60.9)	6659 (60.5)	0.008
Other race^b^	418 (2.6)	305 (2.5)	0.006	281 (2.6)	291 (2.6)	0.006
Primary language English	10359 (64.8)	7967 (65.8)	0.021	7196 (65.4)	7212 (65.5)	0.003
Diagnoses						
Atrial fibrillation or flutter	6197 (38.8)	4540 (37.5)	0.026	4143 (37.6)	4171 (37.9)	0.005
Systolic heart failure	9722 (60.9)	8334 (68.9)	0.169	7431 (67.5)	7380 (67.0)	0.010
Diastolic heart failure	5934 (37.1)	3209 (26.5)	0.229	3151 (28.6)	3158 (28.7)	0.001
Combined heart failure	4066 (25.5)	3370 (27.9)	0.054	2968 (27.0)	3023 (27.5)	0.011
Diabetes mellitus	5934 (37.1)	3209 (26.5)	0.229	3151 (28.6)	3158 (28.7)	0.001
Essential hypertension	11 187 (70.0)	7675 (63.4)	0.140	7215 (65.5)	7196 (65.4)	0.004
Ischemic heart disease	9551 (59.8)	7148 (59.1)	0.014	6592 (59.9)	6572 (59.7)	0.004
Adverse socioeconomic determinants of health^c^	525 (3.3)	387 (3.2)	0.005	352 (3.2)	350 (3.2)	0.001
Medications						
Loop diuretics	11 517 (72.1)	8667 (71.6)	0.010	7818 (71.0)	7846 (71.3)	0.006
β blockers	12 514 (78.3)	9688 (80.1)	0.043	8720 (79.2)	8719 (79.2)	0
Angiotensin II inhibitors	8852 (55.4)	7731 (63.9)	0.174	6788 (61.7)	6762 (61.4)	0.005
Angiotensin converting enzyme inhibitors	4658 (29.2)	3174 (26.2)	0.065	2917 (26.5)	2982 (27.1)	0.013
Potassium sparing diuretics	7155 (44.8)	6246 (51.6)	0.137	5414 (49.2)	5462 (49.6)	0.009
Sacubitril	5258 (32.9)	5471 (45.2)	0.254	4591 (41.7)	4570 (41.5)	0.004
Anti-lipemic agents	10 830 (67.8)	7664 (63.3)	0.094	7139 (64.9)	7142 (64.9)	0.001
Platelet aggregation inhibitors	7934 (49.7)	5975 (49.4)	0.006	5478 (49.8)	5453 (49.5)	0.005
Nitrates	4655 (29.1)	3730 (30.8)	0.037	3365 (30.6)	3355 (30.5)	0.002
Calcium channel blockers	4997 (31.3)	3262 (27.0)	0.095	3078 (28.0)	3069 (27.9)	0.002
Hydralazine	2694 (16.9)	2136 (17.7)	0.021	1894 (17.2)	1875 (17.0)	0.005
Direct renin inhibitors	10 (0.1)	10 (0.1)	0.007	10 (0.1)	10 (0.1)	0
Amiodarone	1587 (9.9)	1352 (11.2)	0.040	1200 (10.9)	1193 (10.8)	0.002
Digoxin	1022 (6.4)	992 (8.2)	0.069	808 (7.3)	831 (7.6)	0.008
Insulins	5102 (31.9)	3306 (27.3)	0.101	3154 (28.7)	3165 (28.8)	0.002
Metformin	3197 (20.0)	1778 (14.7)	0.141	1742 (15.8)	1741 (15.8)	0
Glucagon-like peptide-1 (GLP-1) analogues	1003 (6.3)	587 (4.9)	0.062	557 (5.1)	572 (5.2)	0.006
Dipeptidyl peptidase 4 (DPP-4) inhibitors	759 (4.8)	429 (3.5)	0.060	413 (3.8)	423 (3.8)	0.005
Sulfonylureas	1219 (7.6)	677 (5.6)	0.082	668 (6.1)	667 (6.1)	0
Glomerular filtration rate, mL/min/1.73 m^2^						
0 to <40	4163 (26.1)	3152 (26.1)	0	2910 (26.4)	2882 (26.2)	0.006
40 to <80	10 135 (63.4)	7918 (65.4)	0.042	7170 (65.1)	7141 (64.9)	0.006
80 to <120	5366 (33.6)	4512 (37.3)	0.078	3960 (36.0)	3974 (36.1)	0.003
120 to <150	965 (6.0)	903 (7.5)	0.057	763 (6.9)	749 (6.8)	0.005
≥150	330 (2.1)	311 (2.6)	0.034	266 (2.4)	262 (2.4)	0.002
Hemoglobin A_1c_, %						
0 to <3	10 (0.1)	10 (0.1)	0.007	10 (0.1)	10 (0.1)	0
3 to <6	3175 (19.9)	2596 (21.5)	0.039	2330 (21.2)	2308 (21.0)	0.005
6 to <9	5002 (31.3)	3378 (27.9)	0.074	3188 (29)	3165 (28.8)	0.005
9 to <12	1157 (7.2)	653 (5.4)	0.076	634 (5.8)	639 (5.8)	0.002
≥12	366 (2.3)	205 (1.7)	0.043	209 (1.9)	198 (1.8)	0.007
B-type natriuretic peptide, pg/mL						
0 to <150	1938 (12.1)	1556 (12.9)	0.022	1392 (12.6)	1367 (12.4)	0.007
150 to <300	1334 (8.4)	1077 (8.9)	0.020	961 (8.7)	939 (8.5)	0.007
300 to <450	955 (6.0)	794 (6.6)	0.024	699 (6.4)	689 (6.3)	0.004
450 to <600	684 (4.3)	565 (4.7)	0.019	484 (4.4)	495 (4.5)	0.005
≥600	2298 (14.4)	1868 (15.4)	0.030	1664 (15.1)	1665 (15.1)	0
N-terminal pro-brain natriuretic peptide, pg/mL						
0 to <300	887 (5.6)	679 (5.6)	0.003	607 (5.5)	618 (5.6)	0.004
300 to <600	665 (4.2)	567 (4.7)	0.025	492 (4.5)	488 (4.4)	0.002
600 to <900	560 (3.5)	480 (4.0)	0.024	418 (3.8)	409 (3.7)	0.004
900 to <1200	507 (3.2)	385 (3.2)	0	356 (3.2)	345 (3.1)	0.006
≥1200	2239 (14)	1791 (14.8)	0.022	1645 (14.9)	1629 (14.8)	0.004
Left ventricular ejection fraction, %						
0 to <50	1380 (8.6)	1408 (11.6)	0.099	1204 (10.9)	1181 (10.7)	0.007
≥50	971 (6.1)	599 (5.0)	0.049	601 (5.5)	578 (5.3)	0.009
Hospitalization	7170 (44.9)	5498 (45.4)	0.011	4989 (45.3)	5000 (45.4)	0.002

Abbreviations: SMD, absolute standardized mean difference.

SI conversion factors: To convert B-type natriuretic peptide to ng/L, multiply by 1.0; hemoglobin A_1c_ to proportion of total hemoglobin, multiply by 0.01; N-terminal pro-brain natriuretic peptide, multiply by 1.0.

^a^ To protect patient confidentiality, values of 10 may represent fewer than 10 patients. SMDs less than 0.1 suggest balance of characteristics between exposure groups.

^b^ Other race is defined internally by TriNetX.

^c^ *International Statistical Classification of Diseases and Related Health Problems, Tenth Revision *code Z55-Z65.

**Figure 2.**
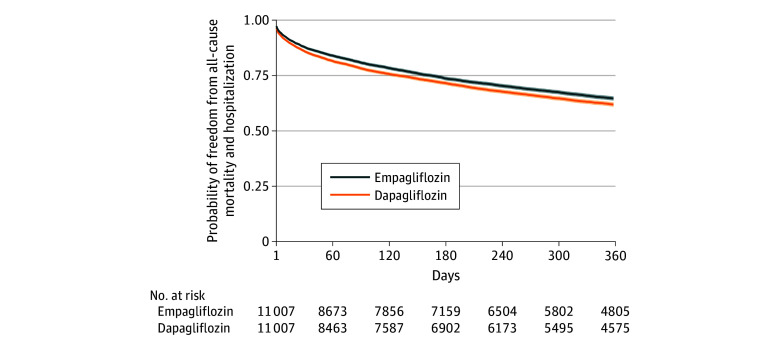
Survival Curve and Associated 95% CIs for the Composite Outcome of All-Cause Mortality or Hospitalization Shaded area indicate 95% CI.

In the 1 year after SGLT2 inhibitor initiation, 3545 patients (32.2%) who received empagliflozin experienced the composite outcome of death or hospitalization vs 3828 (34.8%) of those who received dapagliflozin (HR, 0.90 [95% CI, 0.86-0.94]; log-rank *P* < .001; E-value 1.36) (Figure 2). Similarly, patients who received empagliflozin were less likely to be hospitalized (3270 [29.7%] events vs 3537 [32.1%] events; HR, 0.90 [95% CI, 0.86-0.94]), but all-cause mortality did not differ between exposure groups (691 [6.3%] events vs 764 [6.9%] events; HR, 0.91 [95% CI, 0.82-1.00]) (eFigures 2 and 3 in Supplement 1). A total of 4188 (38.0%) of patients who received empagliflozin and 4422 (40.2%) who received dapagliflozin had at least 1 hemoglobin A_1c_ measurement in the 1 year after SGLT2 inhibitor initiation. Among patients with hemoglobin A_1c_ measurements the mean (SD) of last measured hemoglobin A_1c_ levels was 6.8% (1.6%) in both groups (difference in means *P* = .64) (to convert to proportion of total hemoglobin, multiply by 0.01). In those who received empagliflozin, 652 patients (5.9%) experienced at least 1 adverse event (diabetic ketoacidosis: 40 events [6.1%]; urinary tract infection: 620 events [95.1%]) vs 714 patients (6.5%) among those who received dapagliflozin (diabetic ketoacidosis: 44 events [6.2%]; urinary tract infection: 681 events [95.4%]) (difference in proportions, *P* = .08).

Among those with HFrEF (16 892 patients), characteristics were balanced after matching (eTable 2 in Supplement 1). In the 1-year following SGLT2 inhibitor initiation, patients who received empagliflozin had fewer composite outcome events compared with those who received dapagliflozin (2430 [33.2%] events vs 2598 [35.5%] events; HR, 0.92 [95% CI, 0.87-0.97]) (eFigure 4 in Supplement 1). Similarly, among those with HFpEF (10 911 individuals) (eTable 3 in Supplement 1), patients who received empagliflozin were less likely to experience all-cause mortality or hospitalization compared with those who received dapagliflozin (1332 [34.3%] events vs 1424 [36.7%] events; HR, 0.91 [95% CI, 0.84-0.98]) (eFigure 5 in Supplement 1).

## Discussion

In this multicenter retrospective cohort study that used clinical data, patients who initiated empagliflozin were less likely to experience the composite of all-cause mortality or hospitalization compared with patients who initiated dapagliflozin. These results were determined by differences in rates of hospitalization. Our results suggest that there were possible differences in medication outcomes between specific medications within the SGLT2 inhibitor class. Future studies are needed to clarify potential mechanisms that could explain the observed differences.

Our results should be considered in the context of prior work. A small, single center retrospective study found that empagliflozin (vs dapagliflozin) was associated with greater increases in left ventricular ejection fraction and New York Heart Association Class.^5^ The results of Hao et al^17^ and our own may suggest that there are differences in the degree of cardiac remodeling between different SGLT2 inhibitors. Unlike our results, a recent meta-analysis^1^ of clinical trials showed similar improvements in cardiovascular outcomes between empagliflozin and dapagliflozin compared with placebo. We speculate that the discrepant results between the association and observational outcomes data could be explained by differences in adherence (that were unable to be determined in our study) or differences in comorbidity burden (eg, diabetes) and treatments^4,19^ that could lead to synergistic outcomes of empagliflozin relative to dapagliflozin^4^ or differences in all-cause outcomes associated with heart failure-specific outcomes. Future studies should explore potential mechanisms by which the association and outcomes may differ and directly compare outcomes of empagliflozin to dapagliflozin in a pragmatic comparative effectiveness randomized controlled trial.

### Limitations

This study has limitations. Observational studies are at risk for unmeasured confounding (eg, New York Heart Association functional class); however, the E-value of 1.36 suggests that our findings would only be altered in the presence of an unmeasured confounder with a 30% or greater association with treatment assignment and outcome. Current guidelines^18^ recommend treating patients with heart failure with quadruple therapy, including angiotensin receptor-neprilysin inhibitors, SGLT2 inhibitors, β blockers, and mineralocorticoid receptor antagonists. In our study, use of each component of quadruple therapy ranged from approximately 30% to 80% of patients. The reasons that patients did not receive all components of quadruple therapy is unclear. Our results should not be used as evidence to include empagliflozin over dapagliflozin in quadruple therapy regimens. We were unable to calculate cause-specific mortality or hospitalization due to limitations in the TriNetX platform. TriNetX platform limitations did not allow us to calculate cluster-robust standard errors to account for pair membership or patient practice clustering. Finally, due to the platform limitations, we were unable to determine the specific onset of heart failure in included patients, thus unable to quantify potential risks for immortal time bias.^20^ However, both exposures in this study have the same indications, and inclusion was limited to time after both treatments were available and approved for use in patients with heart failure, all of which likely minimize risks of immortal time.

## Conclusions

In this cohort study using propensity score matching, empagliflozin was associated with lower rates of hospitalization at 1 year compared with dapagliflozin. Future studies are needed confirm these findings and to understand why outcomes may differ from those of efficacy trial meta-analyses.

## Notes

Supplement 1. **eTable 1.** Covariable Definitions **eFigure 1.** Distribution of Propensity Scores Before and After Matching **eFigure 2.** Hospitalization in the 1-Year After SGLT2 Inhibitor Initiation Among Patients With Heart Failure **eFigure 3.** All-Cause Mortality in the 1-Year After SGLT2 Inhibitor Initiation Among Patients With Heart Failure **eTable 2.** Characteristics of Patients With Heart Failure With Reduced Ejection Fraction in the 12-Months Prior to Empagliflozin or Dapagliflozin Initiation **eFigure 4.** All-Cause Mortality or Hospitalization in the 1-Year After SGLT2 Inhibitor Initiation Among Patients With Heart Failure With Reduced Ejection Fraction **eTable 3.** Characteristics of Patients With Heart Failure With Preserved Ejection Fraction in the 12-Months Prior to Empagliflozin or Dapagliflozin Initiation **eFigure 5.** All-Cause or Hospitalization in the 1-Year After SGLT2 Inhibitor Initiation Among Patients With Heart Failure With Preserved Ejection Fraction

Supplement 2.
Data Sharing Statement
