# Error in Results

**DOI:** 10.1001/jamanetworkopen.2024.28489

**Published:** 2024-07-24

In the Original Investigation titled “Comparative Outcomes of Empagliflozin to Dapagliflozin in Patients With Heart Failure,”^1^ published May 2, 2024, there was a typo in the Results section. The text stated that 2828 patients who received dapagliflozin experienced the composite outcome of death or hospitalization; it should have been 3828 patients. This article has been corrected.^1^
