# Supplementary material for: Comparative Outcomes of Empagliflozin to Dapagliflozin in Patients With Heart Failure
Source: JAMA Netw Open. 2024 May 2;7(5):e249305. doi: 10.1001/jamanetworkopen.2024.9305 (PMC11066699; doi:10.1001/jamanetworkopen.2024.9305)
Supplement: Supplement 1. — eTable 1. Covariable Definitions eFigure 1. Distribution of Propensity Scores Before and After Matching eFigure 2. Hospitalization in the 1-Year After SGLT2 Inhibitor Initiation Among Patients With Heart Failure eFigure 3. All-Cause Mortality in the 1-Year After SGLT2 Inhibitor Initiation Among Patients With Heart Failure eTable 2. Characteristics of Patients With Heart Failure With Reduced Ejection Fraction in the 12-Months Prior to Empagliflozin or Dapagliflozin Initiation eFigure 4. All-Cause Mortality or Hospitalization in the 1-Year After SGLT2 Inhibitor Initiation Among Patients With Heart Failure With Reduced Ejection Fraction eTable 3. Characteristics of Patients With Heart Failure With Preserved Ejection Fraction in the 12-Months Prior to Empagliflozin or Dapagliflozin Initiation eFigure 5. All-Cause or Hospitalization in the 1-Year After SGLT2 Inhibitor Initiation Among Patients With Heart Failure With Preserved Ejection Fraction [file jamanetwopen-e249305-s001.pdf]

Supplemental Online Content

Modzelewski KL, Pipilas A, Bosch NA. Comparative outcomes of empagliflozin to dapagliflozin in patients with heart failure. *JAMA Netw Open*. 2024;7(5):e249305. doi:10.1001/jamanetworkopen.2024.9305

eTable 1. Covariable Definitions
eFigure 1. Distribution of Propensity Scores Before and After Matching.
eFigure 2. Hospitalization in the 1-Year After SGLT2 Inhibitor Initiation Among Patients With Heart Failure
eFigure 3. All-Cause Mortality in the 1-Year After SGLT2 Inhibitor Initiation Among Patients With Heart Failure
eTable 2. Characteristics of Patients With Heart Failure With Reduced Ejection Fraction in the 12-Months Prior to Empagliflozin or Dapagliflozin Initiation
eFigure 4. All-Cause Mortality or Hospitalization in the 1-Year After SGLT2 Inhibitor Initiation Among Patients With Heart Failure With Reduced Ejection Fraction
eTable 3. Characteristics of Patients With Heart Failure With Preserved Ejection Fraction in the 12-Months Prior to Empagliflozin or Dapagliflozin Initiation
eFigure 5. All-Cause or Hospitalization in the 1-Year After SGLT2 Inhibitor Initiation Among Patients With Heart Failure With Preserved Ejection Fraction

This supplemental material has been provided by the authors to give readers additional information about their work.

© 2024 Modzelewski KL et al. *JAMA Network Open.*

eTable 1: Covariable definitions

Covariable (ascertained in the 365 days prior to SGLT2i initiation	Definition
Age	TriNetX demographic variable: Age at Index
Sex	TriNetX demographic variable: Sex
Race	TriNetX demographic variable: Race
Primary language English	ISO 639 - eng
Atrial fibrillation or flutter	ICD-10 I48.x
Systolic heart failure	ICD-10 I50.2x
Diastolic heart failure	ICD-10 I50.3x
Combined heart failure	ICD-10 I50.4x
Diabetes mellitus	ICD-10 E08-E13
Essential hypertension	ICD-10 I10.x
Ischemic heart disease	ICD-10 I20-I25
Adverse socioeconomic determinants of health	ICD-10 Z55-Z65
Loop diuretics	VA CV702
Beta blockers	ATC C07
Angiotensin II inhibitors	ATC CV805
Angiotensin converting enzyme inhibitors	ATC CV800
Potassium sparing diuretics	VA CV704
Sacubitril	RxNorm 1656328
Anti-lipemic agents	VA CV350
Platelet aggregation inhibitors	VA BL117
Nitrates	ATC C01DA
Calcium channel blockers	ATC C08
Hydralazine	RxNorm 5470
Direct renin inhibitors	VA CV806

© 2024 Modzelewski KL et al. *JAMA Network Open.*

Amiodarone	RxNorm 703
Digoxin	RxNorm 3407
Insulins	ATC A10A
Metformin	RxNorm 6809
Glucagon-like peptide-1 (GLP-1) analogues	ATC A10BJ
Dipeptidyl peptidase 4 (DPP-4) inhibitors	ATC A10BH
Sulfonylureas	ATC A10BB
Glomerular filtration rate	TriNetX curated laboratory variable: 8001, Glomerular filtration rate/1.73 sq M.predicted [Volume Rate/Area] in Serum, Plasma or Blood by Creatinine-based formula (MDRD)
Hemoglobin A1C	TriNetX curated laboratory variable: 9037, Hemoglobin A1c/Hemoglobin.total in Blood
B-type Natriuretic peptide	TriNetX curated laboratory variable: 9003,<br>Natriuretic peptide B [Mass/volume] in Serum, Plasma or Blood
N-terminal pro-brain natriuretic peptide	TriNetX curated laboratory variable: 9072,<br>Natriuretic peptide.B prohormone N-Terminal [Mass/volume] in Serum, Plasma or Blood
Left ventricular ejection fraction	TriNetX curated laboratory variable: 2003, Left Ventricular Ejection Fraction (LVEF) (%)
Hospitalization	TriNetX visit variable: Visit: Inpatient Encounter

ATC: Anatomical Therapeutic Chemical Classification; ICD-10: International Classification of Diseases, Tenth Revision; SGLT2i: Sodium-Glucose Transport Protein 2 Inhibitors; VA: VA National Drug File.

eFigure 1: Distribution of propensity scores before and after matching.

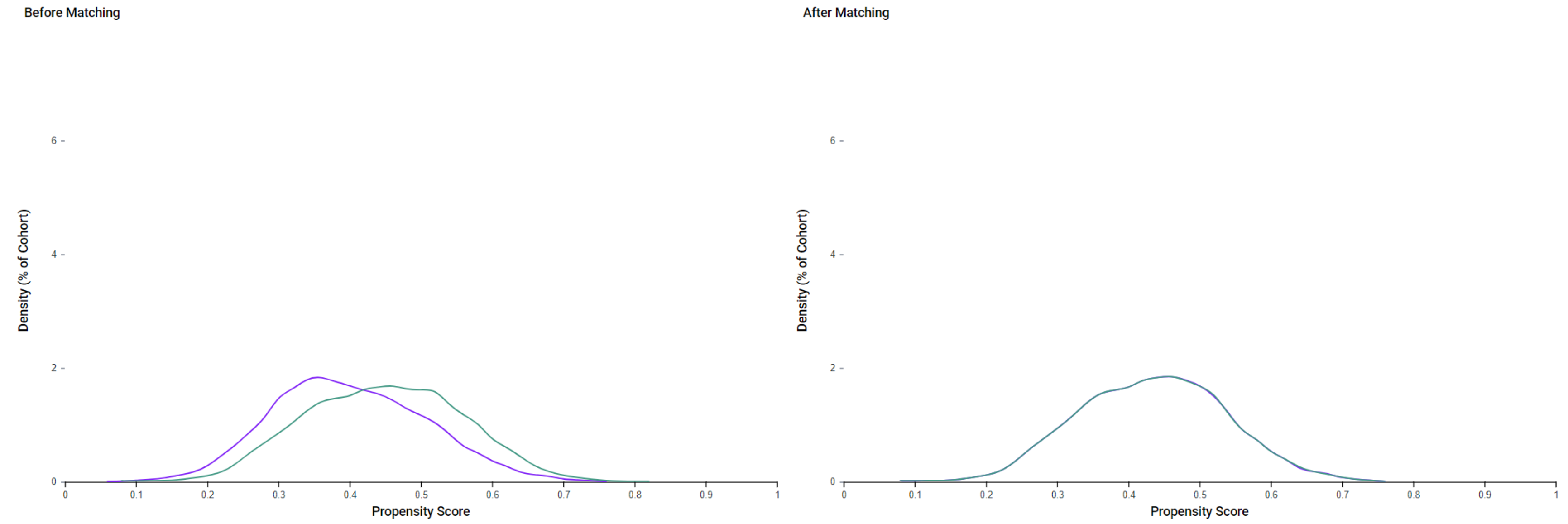

Purple: empagliflozin, Green: dapagliflozin

© 2024 Modzelewski KL et al. *JAMA Network Open.*

eFigure 2: Hospitalization in the 1-year after SGLT2i initiation among patients with heart failure

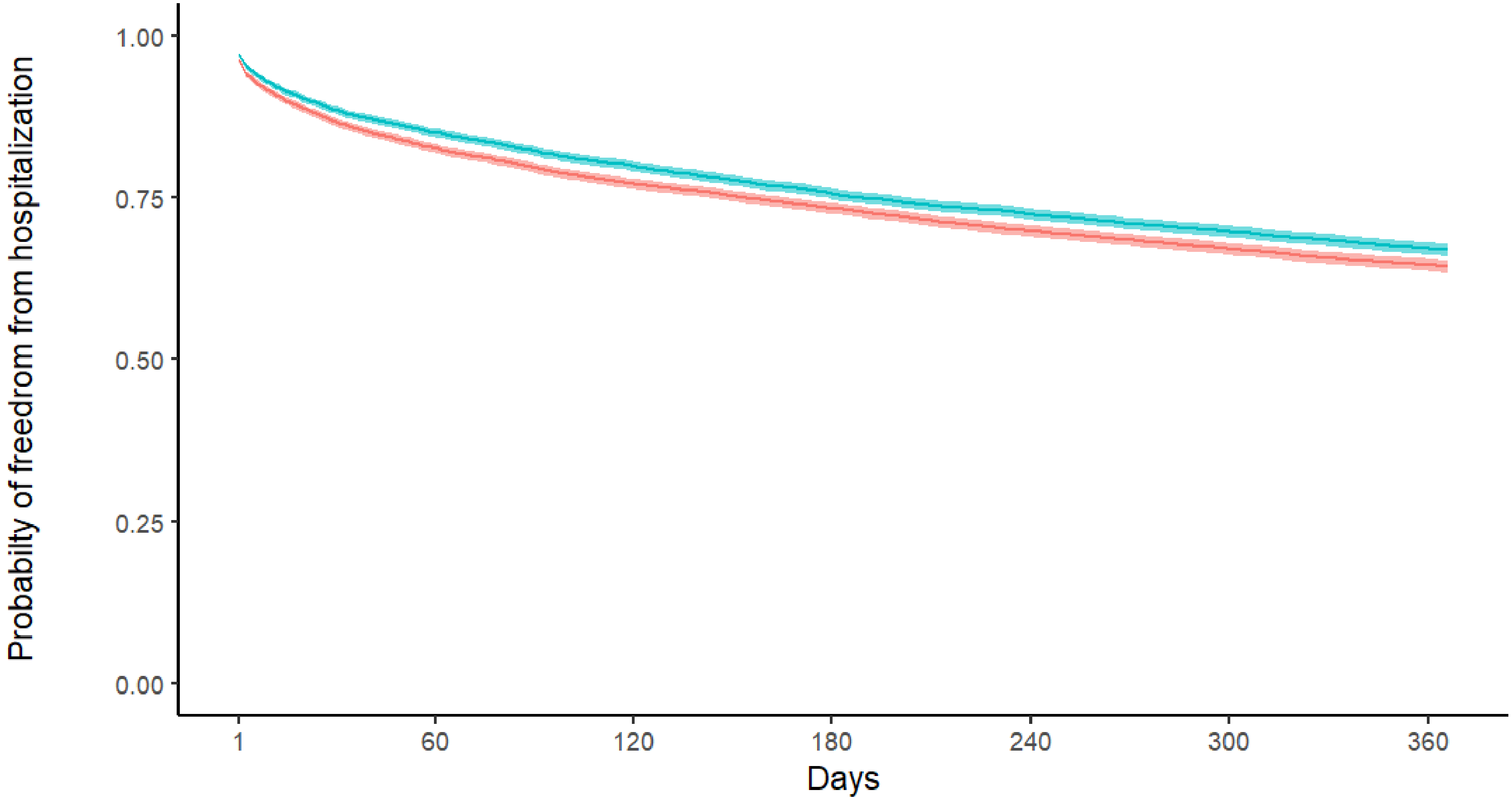

Empagliflozin	11007	8700	7882	7187	6532	5823	4823
Dapagliflozin	11007	8488	7603	6922	6197	5522	4595

Survival curve and associated 95% confidence intervals for hospitalization. Below the curve are shown the number at risk at each time point. Blue: empagliflozin, Red: dapagliflozin

© 2024 Modzelewski KL et al. *JAMA Network Open.*

eFigure 3: All-cause mortality in the 1-year after SGLT2i initiation among patients with heart failure

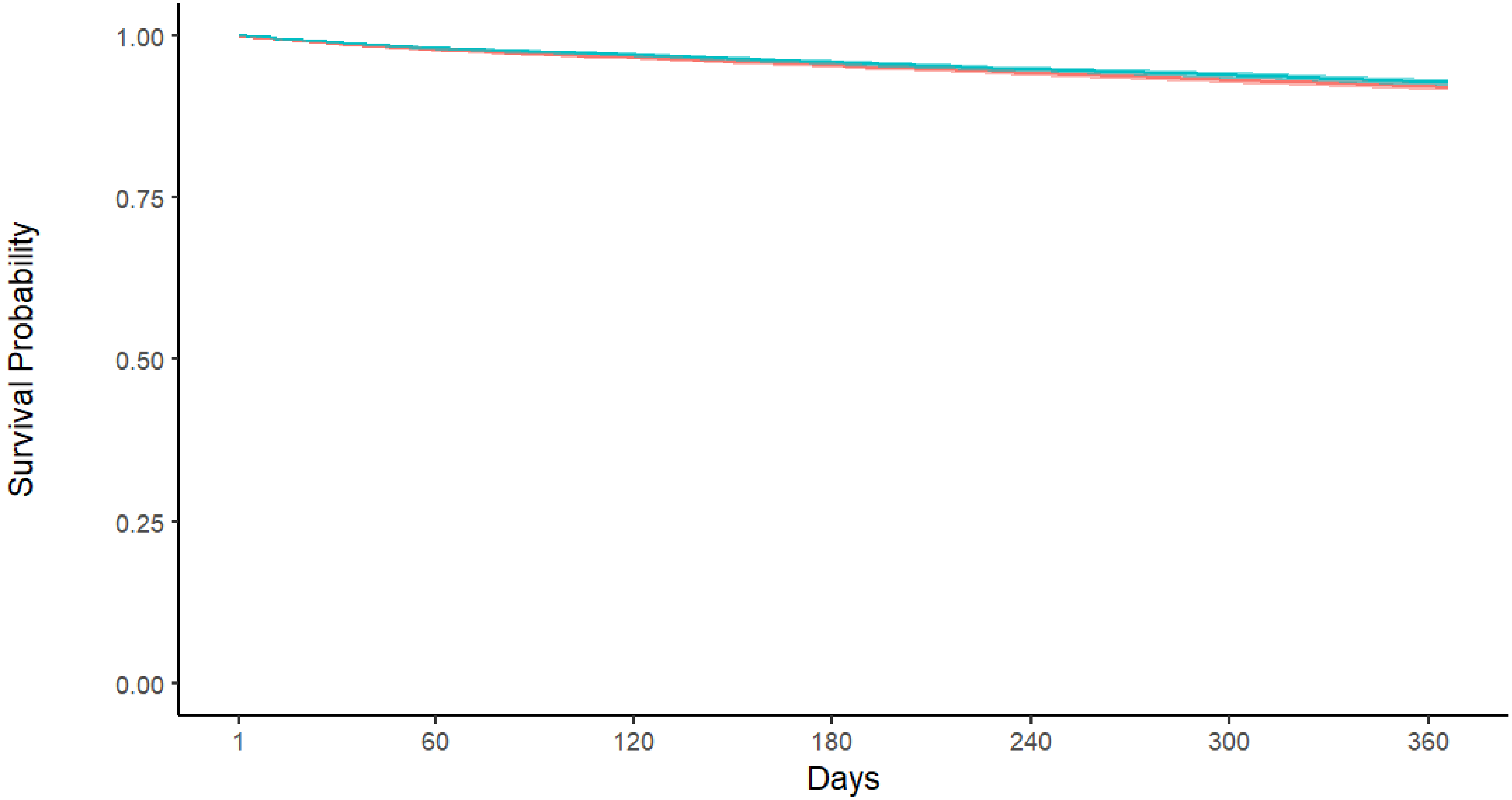

Empagliflozin	11007	10048	9591	9148	8632	7933	6799
Dapagliflozin	11007	10099	9641	9164	8586	7916	6838

Survival curve and associated 95% confidence intervals for the survival. Below the curve are shown the number at risk at each time point. Blue: empagliflozin, Red: dapagliflozin

© 2024 Modzelewski KL et al. *JAMA Network Open.*

eTable 2: Characteristics of patients with heart failure with reduced ejection fraction in the 12-months prior to empagliflozin or dapagliflozin initiation

	Pre-matching			Post-matching		
Characteristic	Empagliflozin (N=9,519)	Dapagliflozin (N=8,287)	SMD	Empagliflozin (N=7,313)	Dapagliflozin (N=7,313)	SMD
Age, mean (SD), years	64.9 (13.5)	62.7 (14.1)	0.162	63.8 (13.7)	63.7 (13.7)	0.007
Sex, No. (%)						
Male sex	6153 (64.6)	5443 (65.7)	0.022	4836 (66.1)	4800 (65.6)	0.010
Female sex	2867 (30.1)	2599 (31.4)	0.027	2261 (30.9)	2269 (31)	0.002
Race, No. (%)						
Asian	356 (3.7)	158 (1.9)	0.111	170 (2.3)	158 (2.2)	0.011
American Indian or Alaska Native	29 (0.3)	29 (0.4)	0.008	25 (0.3)	27 (0.4)	0.005
Black or African American	1937 (20.3)	1718 (20.7)	0.009	1539 (21)	1538 (21)	0.000
Native Hawaiian or Other Pacific Islander	127 (1.3)	33 (0.4)	0.101	34 (0.5)	33 (0.5)	0.002
White	5582 (58.6)	4775 (57.6)	0.021	4420 (60.4)	4383 (59.9)	0.010
Other Race[1]	254 (2.7)	224 (2.7)	0.002	199 (2.7)	210 (2.9)	0.009
Primary language English, No. (%)	6200 (65.1)	5467 (66)	0.018	4787 (65.5)	4813 (65.8)	0.007
Diagnoses, No. (%)						
Atrial fibrillation or flutter	3729 (39.2)	3071 (37.1)	0.044	2801 (38.3)	2763 (37.8)	0.011
Systolic heart failure	8517 (89.5)	7416 (89.5)	0.001	6547 (89.5)	6536 (89.4)	0.005
Diastolic heart failure	1378 (14.5)	1006 (12.1)	0.069	945 (12.9)	959 (13.1)	0.006
Combined heart failure	3512 (36.9)	2979 (35.9)	0.020	2632 (36)	2664 (36.4)	0.009
Diabetes mellitus	4438 (46.6)	3096 (37.4)	0.189	2931 (40.1)	2951 (40.4)	0.006
Essential hypertension	6375 (67)	5116 (61.7)	0.109	4652 (63.6)	4694 (64.2)	0.012
Ischemic heart disease	6265 (65.8)	5128 (61.9)	0.082	4646 (63.5)	4651 (63.6)	0.001
Adverse socioeconomic determinants of health[2]						
Medications, No. (%)						
Loop diuretics	6742 (70.8)	5896 (71.1)	0.007	5179 (70.8)	5194 (71)	0.005
Beta blockers	8027 (84.3)	6970 (84.1)	0.006	6109 (83.5)	6126 (83.8)	0.006
Angiotensin II inhibitors	6214 (65.3)	5869 (70.8)	0.119	5017 (68.6)	5027 (68.7)	0.003
Angiotensin converting enzyme inhibitors	2929 (30.8)	2261 (27.3)	0.077	2046 (28)	2090 (28.6)	0.013
Potassium sparing diuretics	4827 (50.7)	4633 (55.9)	0.104	3931 (53.8)	3945 (53.9)	0.004
Sacubitril	4363 (45.8)	4571 (55.2)	0.187	3792 (51.9)	3793 (51.9)	0.000
Anti-lipemic agents	6541 (68.7)	5207 (62.8)	0.124	4763 (65.1)	4764 (65.1)	0.000
Platelet aggregation inhibitors	5054 (53.1)	4238 (51.1)	0.039	3770 (51.6)	3805 (52)	0.010
Nitrates	3100 (32.6)	2700 (32.6)	0.000	2384 (32.6)	2407 (32.9)	0.007
Calcium channel blockers	2654 (27.9)	1978 (23.9)	0.092	1857 (25.4)	1855 (25.4)	0.001
Hydralazine	1636 (17.2)	1436 (17.3)	0.004	1232 (16.8)	1258 (17.2)	0.009

© 2024 Modzelewski KL et al. *JAMA Network Open.*

	Pre-matching			Post-matching		
Characteristic	Empagliflozin (N=9,519)	Dapagliflozin (N=8,287)	SMD	Empagliflozin (N=7,313)	Dapagliflozin (N=7,313)	SMD
Direct renin inhibitors						
Amiodarone	1158 (12.2)	1024 (12.4)	0.006	881 (12)	899 (12.3)	0.008
Digoxin	753 (7.9)	792 (9.6)	0.058	632 (8.6)	637 (8.7)	0.002
Insulins	2865 (30.1)	2071 (25)	0.114	1915 (26.2)	1947 (26.6)	0.010
Metformin	1696 (17.8)	1071 (12.9)	0.136	1017 (13.9)	1034 (14.1)	0.007
Glucagon-like peptide-1 (GLP-1) analogues	457 (4.8)	307 (3.7)	0.054	295 (4)	294 (4)	0.001
Dipeptidyl peptidase 4 (DPP-4) inhibitors	383 (4)	222 (2.7)	0.075	214 (2.9)	220 (3)	0.005
Sulfonylureas	649 (6.8)	407 (4.9)	0.081	393 (5.4)	396 (5.4)	0.002
Glomerular filtration rate, No. (%)						
0 to <40 mL/min1.73m^2	2345 (24.6)	1985 (24)	0.016	1807 (24.7)	1783 (24.4)	0.008
40 to <80 mL/min/1.73m^2	6105 (64.1)	5486 (66.2)	0.043	4798 (65.6)	4823 (66)	0.007
80 to <120 mL/min/1.73m^2	3406 (35.8)	3259 (39.3)	0.073	2766 (37.8)	2789 (38.1)	0.006
120 to <150 mL/min/1.73m^2	606 (6.4)	638 (7.7)	0.052	519 (7.1)	502 (6.9)	0.009
≥150 mL/min/1.73m2	209 (2.2)	221 (2.7)	0.031	182 (2.5)	176 (2.4)	0.005
Hemoglobin A1C, No. (%)						
0 to <3 %	209 (2.2)	221 (2.7)	0.031	182 (2.5)	176 (2.4)	0.005
3 to <6 %	2005 (21.1)	1879 (22.7)	0.039	1614 (22.1)	1637 (22.4)	0.008
6 to <9 %	2770 (29.1)	2155 (26)	0.069	1981 (27.1)	1974 (27)	0.002
9 to <12 %	643 (6.8)	421 (5.1)	0.071	390 (5.3)	404 (5.5)	0.008
≥12 %	207 (2.2)	130 (1.6)	0.045	119 (1.6)	126 (1.7)	0.007
B-type Natriuretic peptide, No. (%)						
0 to <150 pg/mL	964 (10.1)	939 (11.3)	0.039	788 (10.8)	792 (10.8)	0.002
150 to <300 pg/mL	734 (7.7)	682 (8.2)	0.019	565 (7.7)	601 (8.2)	0.018
300 to <450 pg/mL	572 (6)	530 (6.4)	0.016	459 (6.3)	462 (6.3)	0.002
450 to <600 pg/mL	426 (4.5)	408 (4.9)	0.021	357 (4.9)	350 (4.8)	0.004
≥600 pg/mL	1667 (17.5)	1423 (17.2)	0.009	1245 (17)	1265 (17.3)	0.007
N-terminal pro-brain natriuretic peptide, No. (%)						
0 to <300 pg/mL	466 (4.9)	427 (5.2)	0.012	385 (5.3)	380 (5.2)	0.003
300 to <600 pg/mL	408 (4.3)	408 (4.9)	0.030	349 (4.8)	335 (4.6)	0.009
600 to <900 pg/mL	332 (3.5)	346 (4.2)	0.036	287 (3.9)	271 (3.7)	0.011
900 to <1200 pg/mL	298 (3.1)	263 (3.2)	0.002	246 (3.4)	233 (3.2)	0.010
≥1200 pg/mL	1540 (16.2)	1388 (16.7)	0.015	1231 (16.8)	1224 (16.7)	0.003
Left ventricular ejection fraction No. (%)						
0 to <50%	1187 (12.5)	1187 (14.3)	0.054	1011 (13.8)	1030 (14.1)	0.007
≥50 %	323 (3.4)	223 (2.7)	0.041	222 (3)	210 (2.9)	0.010
Hospitalization, No. (%)	4538 (47.7)	3864 (46.6)	0.021	3434 (47)	3458 (47.3)	0.007

© 2024 Modzelewski KL et al. *JAMA Network Open.*

When a continuous laboratory measure was categorical, the categories were specified as ranges that the continuous laboratory variable could take (e.g., having a value for a glomerular filtration rate 40 to <80 mL/min/1.73m2). In the propensity score model, each category (for both originally continuous and categorical data) became a distinct covariate (with values of either 0 for “not present” or 1 for “is present”). This approach allows all patients to be included in propensity score modeling even in the presence of missing data. Similarly, if a patient had multiple laboratory assessments in the 12-months prior to SGLT2i initiation, it is possible that the patient could have multiple categories coded as 1. Thus, categories for continuous laboratory measures in the table may not sum to the total number of cohort patients (either because patients may not have a value for the measure or because patients may have multiple values for the measure). To protect patient confidentiality, values of 10 may represent fewer than 10 patients. SMD: absolute standardized mean difference – SMDs less than 0.1 suggest balance of characteristics between exposure groups. [1]Other Race is defined internally by TriNetX. [2]International Classification of Diseases, Tenth Revision Codes Z55-Z65.

© 2024 Modzelewski KL et al. *JAMA Network Open.*

eFigure 4: All-cause or hospitalization in the 1-year after SGLT2i initiation among patients with heart failure with reduced ejection fraction.

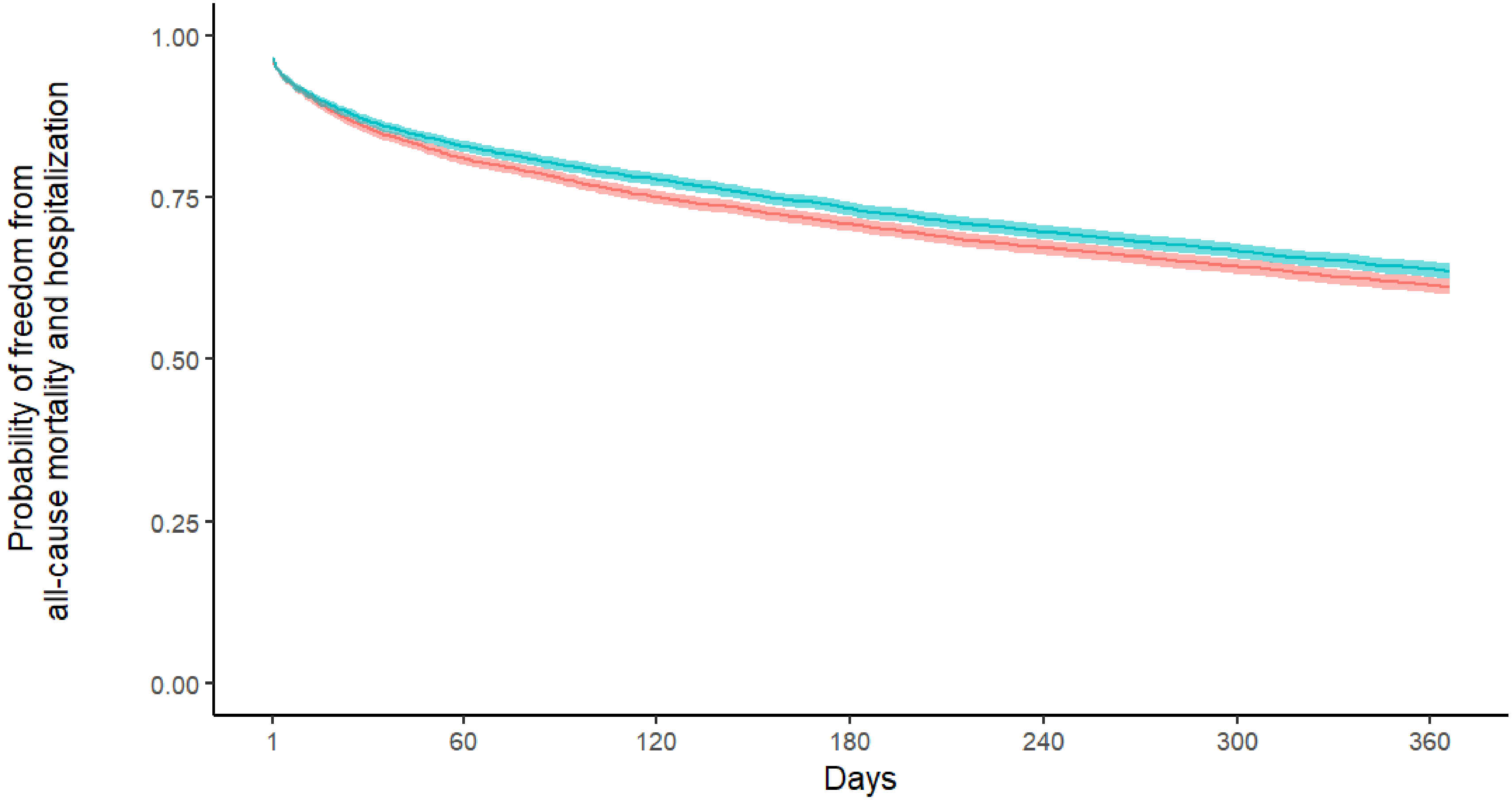

Empagliflozin	7313	5714	5183	4736	4326	3855	3204
Dapagliflozin	7313	5609	5020	4569	4116	3690	3066

Survival curve and associated 95% confidence intervals for the composite outcome of all-cause mortality or hospitalization. Below the curve are shown the number at risk at each time point. Blue: empagliflozin, Red: dapagliflozin

© 2024 Modzelewski KL et al. *JAMA Network Open.*

eTable 3: Characteristics of patients with heart failure with preserved ejection fraction in the 12-months prior to empagliflozin or dapagliflozin initiation

	Pre-matching			Post-matching		
Characteristic	Empagliflozin (N=6,790)	Dapagliflozin (N=4,121)	SMD	Empagliflozin (N=3,883)	Dapagliflozin (N=3,883)	SMD
Age, mean (SD), years	68.3 (13.0)	66.1 (13.8)	0.162	66.8 (13.6)	66.7 (13.6)	0.001
Sex, No. (%)						
Male sex	3367 (49.6)	2234 (54.2)	0.093	2063 (53.1)	2066 (53.2)	0.002
Female sex	3007 (44.3)	1721 (41.8)	0.051	1655 (42.6)	1651 (42.5)	0.002
Race, No. (%)						
Asian	227 (3.3)	84 (2)	0.081	71 (1.8)	84 (2.2)	0.024
American Indian or Alaska Native	13 (0.2)	12 (0.3)	0.020	10 (0.3)	10 (0.3)	0.000
Black or African American	1266 (18.6)	806 (19.6)	0.023	743 (19.1)	740 (19.1)	0.002
Native Hawaiian or Other Pacific Islander	77 (1.1)	15 (0.4)	0.089	18 (0.5)	15 (0.4)	0.012
White	4235 (62.4)	2629 (63.8)	0.030	2510 (64.6)	2505 (64.5)	0.003
Other Race[1]	165 (2.4)	103 (2.5)	0.004	101 (2.6)	95 (2.4)	0.010
Primary language English, No. (%)	4517 (66.5)	2841 (68.9)	0.052	2682 (69.1)	2652 (68.3)	0.017
Diagnoses, No. (%)						
Atrial fibrillation or flutter	2812 (41.4)	1746 (42.4)	0.019	1681 (43.3)	1646 (42.4)	0.018
Systolic heart failure	2149 (31.6)	1597 (38.8)	0.149	1450 (37.3)	1453 (37.4)	0.002
Diastolic heart failure	4885 (71.9)	2427 (58.9)	0.277	2423 (62.4)	2398 (61.8)	0.013
Combined heart failure	2699 (39.8)	2212 (53.7)	0.282	1964 (50.6)	1980 (51)	0.008
Diabetes mellitus	3669 (54.0)	1931 (46.9)	0.144	1873 (48.2)	1881 (48.4)	0.004
Essential hypertension	5210 (76.7)	2945 (71.5)	0.120	2826 (72.8)	2804 (72.2)	0.013
Ischemic heart disease	3825 (56.3)	2361 (57.3)	0.019	2229 (57.4)	2229 (57.4)	0.000
Adverse socioeconomic determinants of health[2]	258 (3.8)	136 (3.3)	0.027	132 (3.4)	130 (3.3)	0.003
Medications, No. (%)						
Loop diuretics	5161 (76.0)	3130 (76)	0.001	2977 (76.7)	2946 (75.9)	0.019
Beta blockers	4978 (73.3)	3073 (74.6)	0.029	2890 (74.4)	2872 (74.0)	0.011
Angiotensin II inhibitors	3121 (46.0)	2261 (54.9)	0.179	2057 (53.0)	2045 (52.7)	0.006
Angiotensin converting enzyme inhibitors	1911 (28.1)	1012 (24.6)	0.081	981 (25.3)	972 (25.0)	0.005
Potassium sparing diuretics	2838 (41.8)	1994 (48.4)	0.133	1856 (47.8)	1824 (47.0)	0.017
Sacubitril	1380 (20.3)	1331 (32.3)	0.274	1134 (29.2)	1132 (29.2)	0.001
Anti-lipemic agents	4568 (67.3)	2621 (63.6)	0.077	2509 (64.6)	2481 (63.9)	0.015
Platelet aggregation inhibitors	3188 (47)	1971 (47.8)	0.018	1866 (48.1)	1843 (47.5)	0.012
Nitrates	1843 (27.1)	1241 (30.1)	0.066	1153 (29.7)	1144 (29.5)	0.005
Calcium channel blockers	2430 (35.8)	1288 (31.3)	0.096	1263 (32.5)	1247 (32.1)	0.009
Hydralazine	1235 (18.2)	791 (19.2)	0.026	735 (18.9)	732 (18.9)	0.002

© 2024 Modzelewski KL et al. *JAMA Network Open.*

	Pre-matching			Post-matching		
Characteristic	Empagliflozin (N=6,790)	Dapagliflozin (N=4,121)	SMD	Empagliflozin (N=3,883)	Dapagliflozin (N=3,883)	SMD
Direct renin inhibitors						
Amiodarone	539 (7.9)	407 (9.9)	0.068	383 (9.9)	370 (9.5)	0.011
Digoxin	381 (5.6)	318 (7.7)	0.084	276 (7.1)	275 (7.1)	0.001
Insulins	2266 (33.4)	1225 (29.7)	0.079	1171 (30.2)	1184 (30.5)	0.007
Metformin	1358 (20.0)	618 (15.0)	0.132	621 (16.0)	612 (15.8)	0.006
Glucagon-like peptide-1 (GLP-1) analogues	512 (7.5)	230 (5.6)	0.079	227 (5.8)	228 (5.9)	0.001
Dipeptidyl peptidase 4 (DPP-4) inhibitors	318 (4.7)	152 (3.7)	0.050	142 (3.7)	147 (3.8)	0.007
Sulfonylureas	536 (7.9)	239 (5.8)	0.083	221 (5.7)	237 (6.1)	0.017
Glomerular filtration rate, No. (%)						
0 to <40 mL/min1.73m^2	1974 (29.1)	1210 (29.4)	0.006	1159 (29.8)	1148 (29.6)	0.006
40 to <80 mL/min/1.73m^2	4418 (65.1)	2734 (66.3)	0.027	2554 (65.8)	2568 (66.1)	0.008
80 to <120 mL/min/1.73m^2	2117 (31.2)	1402 (34)	0.061	1299 (33.5)	1308 (33.7)	0.005
120 to <150 mL/min/1.73m^2	369 (5.4)	261 (6.3)	0.038	234 (6)	240 (6.2)	0.006
≥150 mL/min/1.73m2	126 (1.9)	107 (2.6)	0.050	93 (2.4)	97 (2.5)	0.007
Hemoglobin A1C, No. (%)						
0 to <3 %	0 (0)	10 (0.2)	0.070	0 (0)	0 (0)	0
3 to <6 %	1358 (20)	930 (22.6)	0.063	854 (22)	858 (22.1)	0.002
6 to <9 %	2279 (33.6)	1233 (29.9)	0.078	1214 (31.3)	1188 (30.6)	0.014
9 to <12 %	485 (7.1)	226 (5.5)	0.068	220 (5.7)	223 (5.7)	0.003
≥12 %	147 (2.2)	83 (2.0)	0.011	78 (2.0)	80 (2.1)	0.004
B-type Natriuretic peptide, No. (%)						
0 to <150 pg/mL	1008 (14.8)	595 (14.4)	0.012	578 (14.9)	558 (14.4)	0.015
150 to <300 pg/mL	658 (9.7)	409 (9.9)	0.008	385 (9.9)	385 (9.9)	0.000
300 to <450 pg/mL	461 (6.8)	313 (7.6)	0.031	278 (7.2)	296 (7.6)	0.018
450 to <600 pg/mL	322 (4.7)	202 (4.9)	0.007	188 (4.8)	190 (4.9)	0.002
≥600 pg/mL	982 (14.5)	637 (15.5)	0.028	593 (15.3)	602 (15.5)	0.006
N-terminal pro-brain natriuretic peptide, No. (%)						
0 to <300 pg/mL	482 (7.1)	322 (7.8)	0.027	300 (7.7)	298 (7.7)	0.002
300 to <600 pg/mL	305 (4.5)	248 (6.0)	0.068	220 (5.7)	211 (5.4)	0.010
600 to <900 pg/mL	273 (4.0)	184 (4.5)	0.022	156 (4.0)	167 (4.3)	0.014
900 to <1200 pg/mL	845 (12.4)	597 (14.5)	0.060	538 (13.9)	548 (14.1)	0.007
≥1200 pg/mL	845 (12.4)	597 (14.5)	0.060	538 (13.9)	548 (14.1)	0.007
Left ventricular ejection fraction No. (%)						
0 to <50%	265 (3.9)	270 (6.6)	0.119	223 (5.7)	227 (5.8)	0.004
≥50 %	658 (9.7)	366 (8.9)	0.028	383 (9.9)	362 (9.3)	0.018
Hospitalization, No. (%)	3042 (44.8)	1866 (45.3)	0.010	1771 (45.6)	1764 (45.4)	0.004

When a continuous laboratory measure was categorical, the categories were specified as ranges that the continuous laboratory variable could take (e.g., having a value for a glomerular filtration rate 40 to <80 mL/min/1.73m2). In the propensity score model, each category (for both originally continuous and categorical data) became a distinct covariate (with values of either 0 for “not present” or 1 for “is present”). This approach allows all patients to be included in propensity score modeling even in the presence of missing data. Similarly, if a patient had multiple laboratory assessments in the 12-months prior to SGLT2i initiation, it is possible that the patient could have multiple categories coded as 1. Thus, categories for continuous laboratory measures in the table may not sum to the total number of cohort patients (either because patients may not have a value for the measure or because patients may have multiple values for the measure). To protect patient confidentiality, values of 10 may represent fewer than 10 patients. SMD: absolute standardized mean difference – SMDs less than 0.1 suggest balance of characteristics between exposure groups. [1]Other Race is defined internally by TriNetX. [2]International Classification of Diseases, Tenth Revision Codes Z55-Z65.

© 2024 Modzelewski KL et al. *JAMA Network Open.*

eFigure 5: All-cause or hospitalization in the 1-year after SGLT2i initiation among patients with heart failure with preserved ejection fraction.

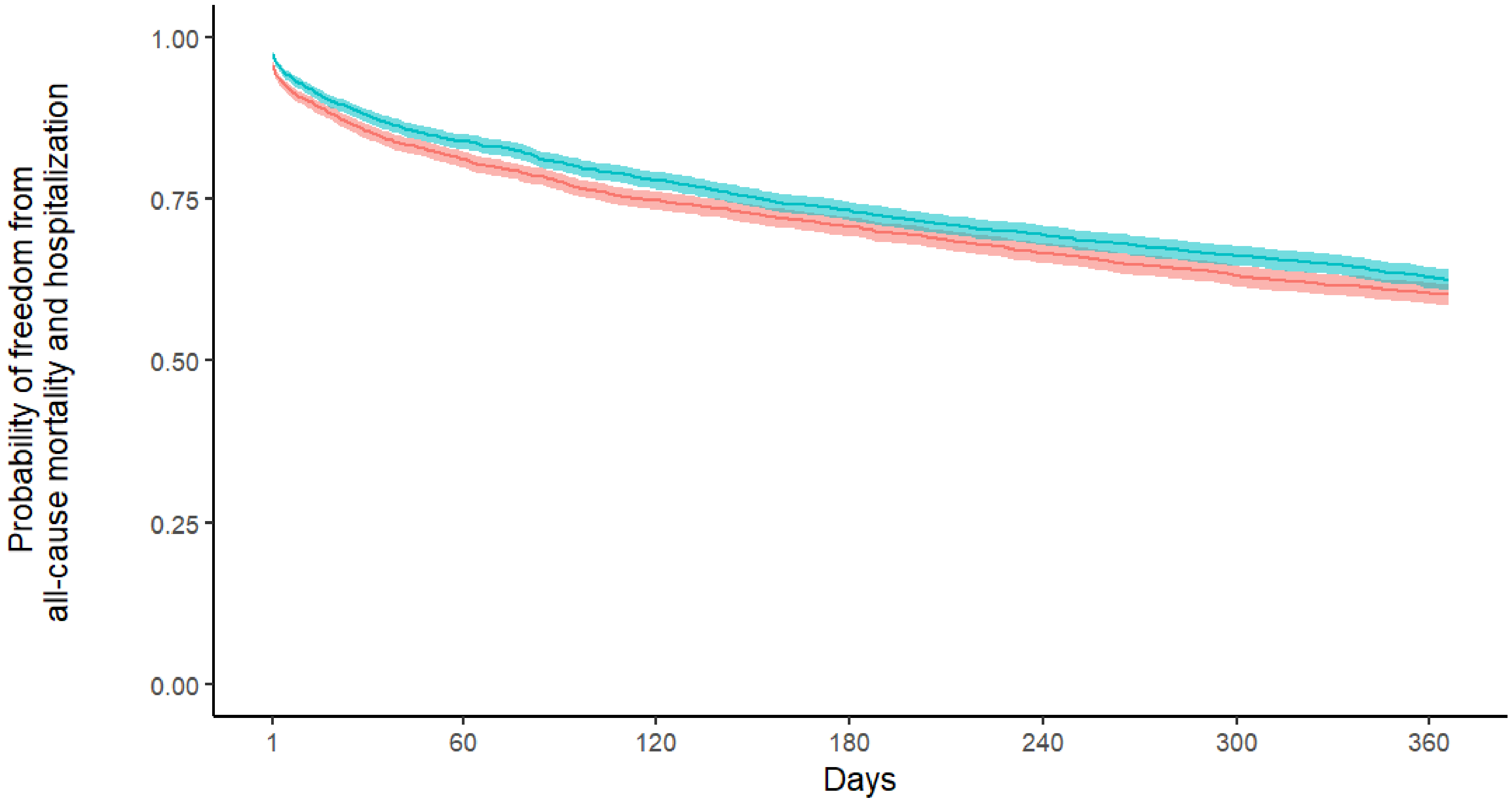

Empagliflozin	3883	3092	2791	2548	2314	2068	1724
Dapagliflozin	3883	2999	2681	2448	2205	1931	1605

Survival curve and associated 95% confidence intervals for the composite outcome of all-cause mortality or hospitalization. Below the curve are shown the number at risk at each time point. Blue: empagliflozin, Red: dapagliflozin

© 2024 Modzelewski KL et al. *JAMA Network Open.*
